# Towards understanding the messengers of extracellular space: Computational models of outside-in integrin reaction networks

**DOI:** 10.1016/j.csbj.2020.12.025

**Published:** 2020-12-29

**Authors:** Zeynep Karagöz, Laura Rijns, Patricia Y.W. Dankers, Martijn van Griensven, Aurélie Carlier

**Affiliations:** a Department of Cell Biology-Inspired Tissue Engineering, MERLN Institute for Technology-Inspired Regenerative Medicine, Maastricht University, Universiteitssingel 40, 6229 ER Maastricht, the Netherlands; b Department of Biomedical Engineering and Institute for Complex Molecular Systems, Eindhoven University of Technology, PO Box 513, 5600 MB Eindhoven, the Netherlands

## Abstract

The interactions between cells and their extracellular matrix (ECM) are critically important for homeostatic control of cell growth, proliferation, differentiation and apoptosis. Transmembrane integrin molecules facilitate the communication between ECM and the cell. Since the characterization of integrins in the late 1980s, there has been great advancement in understanding the function of integrins at different subcellular levels. However, the versatility in molecular pathways integrins are involved in, the high diversity in their interaction partners both outside and inside the cell as well as on the cell membrane and the short lifetime of events happening at the cell–ECM interface make it difficult to elucidate all the details regarding integrin function experimentally. To overcome the experimental challenges and advance the understanding of integrin biology, computational modeling tools have been used extensively. In this review, we summarize the computational models of integrin signaling while we explain the function of integrins at three main subcellular levels (outside the cell, cell membrane, cytosol). We also discuss how these computational modeling efforts can be helpful in other disciplines such as biomaterial design. As such, this review is a didactic modeling summary for biomaterial researchers interested in complementing their experimental work with computational tools or for seasoned computational scientists that would like to advance current *in silico* integrin models.

## Graphical abstract

**Figure:**
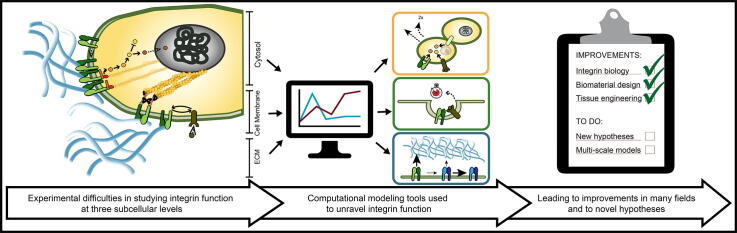

## 1 Introduction

The evolution of cell adhesion, both to other cells and to surfaces, has been a critical step in the emergence of multicellular organisms on earth [1]. Today, we know that cells of all multicellular metazoans, reside in a mesh of fibrous proteins, referred to as the extracellular matrix (ECM) [2]. Adhesion to the ECM is required for the homeostatic control of cell growth, proliferation, differentiation and apoptosis [3]. Furthermore, interaction between cells of the same tissue/organ is facilitated by the ECM, resulting in biochemical and biophysical information exchange [4]. When the natural cell–ECM interaction is perturbed, because cells cannot adhere or the ECM properties have drastically changed, morbid or cancerous phenotypes are observed at the cell/tissue/organ level [5], [6], [7]. The ECM therefore, not only functions as a structural support for a group of cells in a tissue, but it actively communicates with the cells to ensure homeostasis.

**Fig. 1:**
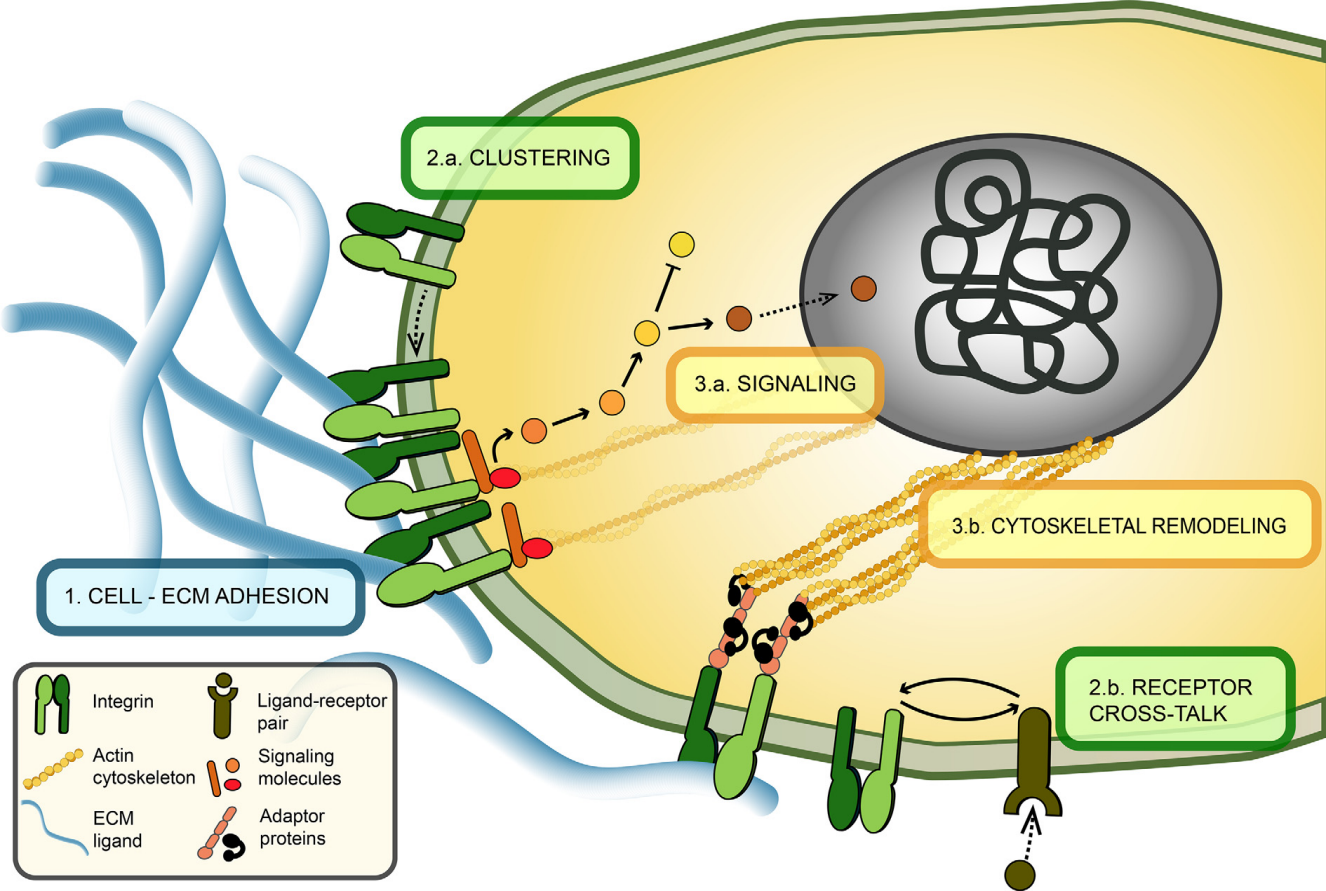
Integrins are transmembrane proteins that function at multiple cellular levels. Outside the cells (1, blue), ectodomains of integrins selectively bind to extracellular ligands. On the cell membrane (2, green), multiple integrin molecules are recruited to the focal adhesion site and physically cluster together (2.a) and/or integrins interact with other cell surface receptors to enhance their activity, resulting in signaling crosstalk. (2.b). In the cytosol (3, yellow), integrins initiate signaling cascades (3.a) and are connected to the actin cytoskeleton via adaptor proteins and can initiate cytoskeletal remodeling (3.b). (For interpretation of the references to colour in this figure legend, the reader is referred to the web version of this article.)

**Fig. 2:**
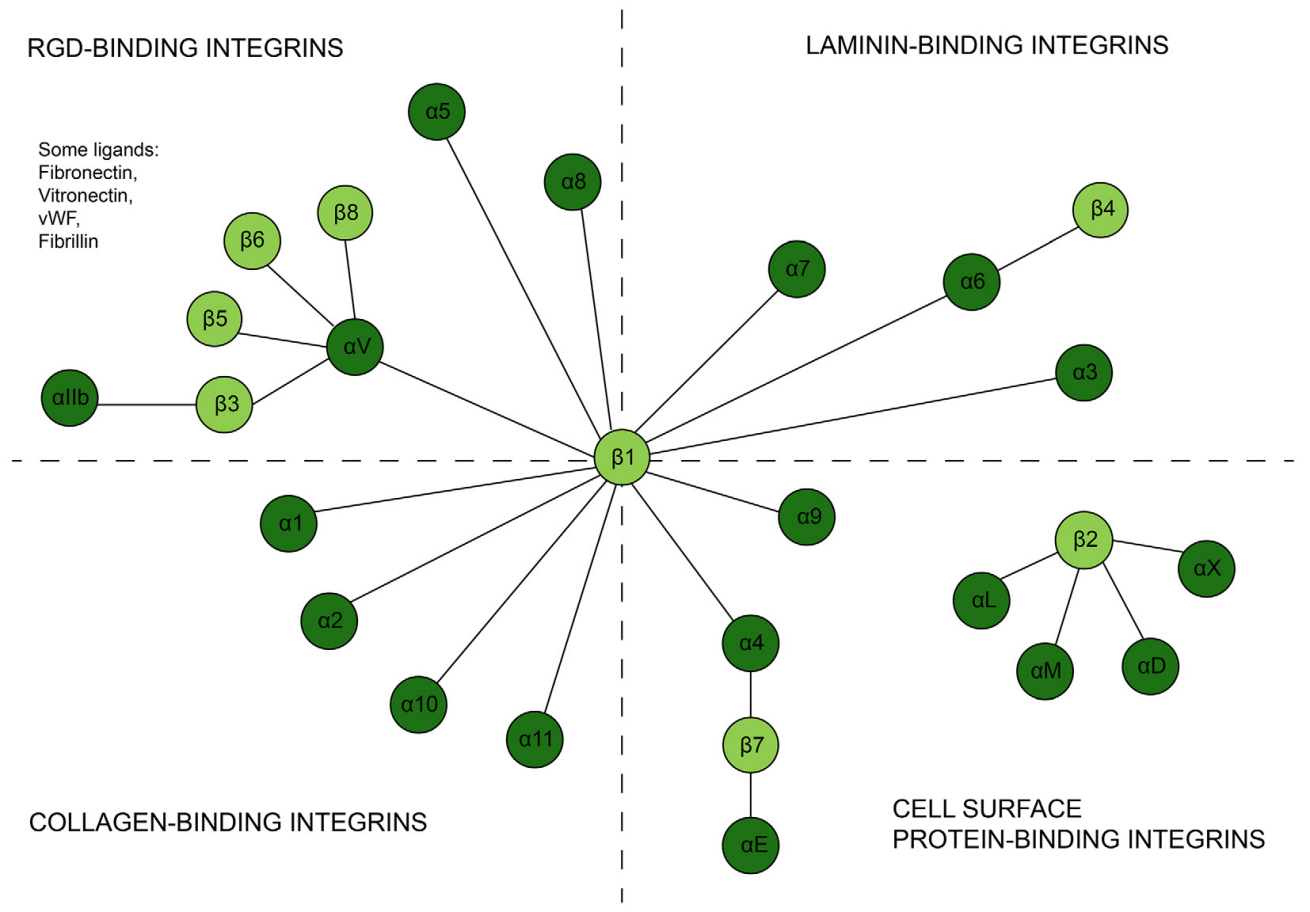
A schematic overview of the 24 unique types of integrins, that are composed of 18 different α (dark green) and eight different β subunits (light green). Integrins are grouped into four classes according to their ligand-binding properties. Adapted from (Hudson et al. 2017). (For interpretation of the references to colour in this figure legend, the reader is referred to the web version of this article.)

Experimental research on the subcellular structures that form the link between cells and their matrix started in the early 1970s [2]. After nearly 20 years, a family of heterodimeric proteins, called integrins, were characterized as cell-surface receptors for ECM proteins that mediate the communication of cells and their ECM in animals [9] (Fig. 1). Each integrin molecule consists of non-covalently associated α and β subunits. To date, 24 unique integrins have been found in mammals, which are combinations of 18 different α and eight different β subunits [10], [11] (Fig. 2). Each integrin molecule is able to recognize and bind to a defined set of ECM ligands via its ectodomain [11], [12] and to cytosolic ligands via its cytoplasmic tails [11]. This way, integrins create physical anchor points between the extracellular space and the cytoplasm (Fig. 1.1, blue).

There are different ways in which integrins orchestrate the communication between the cells and their extracellular environment. Multiple integrins of the same or different type, when bound to their ligands, can cluster together (Fig. 1.2a, green) and initiate the formation of a multi-protein complex at the cell–ECM interface called a “focal adhesion” (FA) [13]. The cytoplasmic side of the FAs contain many different molecules and protein kinases, such as focal adhesion kinase (FAK), proto-oncogene tyrosine-protein kinase Src and small GTPase Ras, to start and maintain several signaling cascades [13]. It is also known that integrins can facilitate and/or enhance signaling via other cell-surface receptors (such as syndecans or receptor tyrosine kinases) by establishing a “cross-talk” with them [14], [15] (Fig. 1.2b, green). In addition, on the cytosolic side, FAs harbor numerous other proteins such as talin and vinculin. Via these proteins, FAs are bound to the actin cytoskeleton and can affect the cell shape and motility [13], [16] (Fig. 1.3b, yellow).

Having such key functions in the cell–ECM communication and initiating cellular responses to signals from the ECM, integrins have been a common target in the biomaterial and tissue engineering fields. Biomaterials designed for regenerative medicine and in specific tissue engineering applications are aimed to direct specific cellular behavior (e.g. regeneration) by designing instructive biomaterials with or without the addition of growth factors [17], [18], [19]. Materials inspired by tissue-specific geometric, chemical and physical properties of the ECM have been successfully used to guide the cells to a desired behavior [18]. Recently, materials decorated with bioactive molecules have been produced to actively communicate with cells [17]. These promising strategies require thorough understanding of integrin function, as it is the key mediator of ECM–cell interactions.

Since the characterization of integrins in the late 1980s, there has been great advancement in understanding the function of integrins at different levels. However, the versatility in molecular pathways integrins are involved in, the high diversity in their interaction partners both outside and inside the cell as well as on the cell membrane and the short lifetime of events happening at the cell–ECM interface make it difficult to elucidate all the details regarding integrin function experimentally [2], [20], [21], [22].

To overcome the experimental difficulties and to integrate knowledge on integrin function at different cellular levels that come from different *in vitro* methods, *in silico* efforts have come into play. Computational modeling of biological data is a powerful tool and helps to resolve complex interactions within biological systems. A computational (or *in silico*) model is a mathematical simplification of the actual system. It aims at replicating the behavior of the system it represents, allowing to perform simulations and test novel hypothesis *in silico* [23], [24].

The level of detail and precision of a computational model, as well as the amount of data needed to build one, depend on the research question and the mathematical method [23]. The most detailed mathematical description of a biological system is by ordinary or partial differential equations (ODEs/PDEs). This type of model provides information on the changes in the amount of each component in the model over time. Although they are precise in the information they provide, these models are parameter-intensive, meaning that one needs the initial amounts of each species observed in the model, as well as the time-dependent relationship between them (e.g. reaction rates) to build the model. These type of models may get very complicated very quickly because every species in the system must be represented by one equation [23]. Logic-based Boolean models are at the other side of the spectrum in terms of precision and data intensity. They are not based on precise measurements of biological molecules, but they work in an ON/OFF manner, based on observations such as “when molecule A is present in the system, B gets activated”. The simplicity in construction makes these type of models suitable for representation of large biological networks such as signaling cascades [23]. The criteria for deciding on a type of model are therefore 1) the amount and the characteristics of the data at hand and 2) the specific purposes of modeling. There can be multiple ways to model a biological process, and all of them can be correct at the same time [25].

Our molecule of interest in this review, integrin, has also been studied *in silico* since it is at the heart of the cell–ECM communication, yet difficult to study experimentally. As integrin function can be divided into three main spatial categories—1) extracellular space (Fig. 1 – blue), 2) cell membrane (Fig. 1 – green) and 3) cytosol (Fig. 1 – yellow)—the computational models of integrin function can also be grouped into the same three main categories. In the following sections, we explain the function of integrin at these three spatial categories following an outside-in perspective and summarize the computational models that belong to each category (Table 1).

**Table 1:** In silico models describing integrin function at different sub-cellular levels that are reviewed in the text. Models are listed under the sub-cellular space category they address.

1. Outside	2. Membrane		3. Cytosol	
Cell – ECM adhesion	Integrin clustering	Receptor cross-talk	Signaling	Cytoskeletal remodeling
Cóndor et al. 2017	Jamali et al. 2012	Bazzazi et al. 2018	Cirit et al. 2010	Macdonald et al. 2008
Blucher et al. 2014	Yu et al. 2017	Bauer et al. 2010		Escribano et al. 2014
Hudson et al. 2017	Cheng et al. 2020			
Bidone et al. 2019				

A review of computational models that centralizes around the molecular reaction networks of integrins does not exist to our knowledge. We aim at filling this gap and providing the scientific community with a guideline that can be used in two ways: first, as a roadmap on how the integrin function can be modeled computationally using different methods, and second, as a starting point for new computational models as we also state possible extensions to existing models and open questions in the field. We do not include in this review the computational models of processes related to integrin function (e.g. mechanosensing and cell motility) that do not explicitly explain the role of integrins. Comprehensive reviews of computational models of mechanosensing [26] and cell shape and motility [27] can be found elsewhere.

## 2 Extracellular matrix proteins binding to integrins

In its simplest form, ligand binding at the interface between the α and β subunits of integrins requires that there are integrins present on the portion of the cell membrane that is exposed to the ECM and that those integrins are in their active form. Such a system has the following reactions:

(Rxn. 1) $$S\overset{k_{D}^{+}/k_{D}^{-}}{↔}I$$

(Rxn. 2) $$L+I\overset{k_{L}^{+}/k_{L}^{-}}{↔}LI$$

where *S* is the inactive integrin concentration that and becomes active with the rate $k_{D}^{+}$. I is the active integrin concentration at the reaction site that can bind to ligands, L, with the reaction rate $k_{L}^{+}$ to form the ligand–integrin complex, LI. The reverse reactions have the rates $k_{D}^{-}$ and $k_{L}^{-}$, respectively.

The rate of change in concentration of each species in this simple system can be expressed as ODEs in the following form:

(1) $$\frac{d\left[I\right]}{\mathrm{dt}}=k_{D}^{+}\left[S\right]-k_{L}^{+}\left[L\right]\left[I\right]+k_{L}^{-}\left[LI\right]-k_{D}^{-}\left[S\right]$$

(2) $$\frac{d\left[L\right]}{\mathrm{dt}}=-k_{L}^{+}\left[L\right]\left[I\right]+k_{L}^{-}\left[LI\right]$$

(3) $$\frac{d\left[S\right]}{\mathrm{dt}}=-k_{D}^{+}\left[S\right]+k_{D}^{-}\left[I\right]$$

By numerically solving these ODEs, some important questions can be answered, such as “What is the equilibrium concentration of each species?”, “How fast does the system reach the equilibrium?” and “How do the equilibrium concentrations depend on the binding parameter values?” To numerically solve a system like this, one needs to plug in the initial concentrations of each species and rate constants in the equations. The system of equations can be converted to computer code and then numerically solved for convenience. Tellurium [28] and bioCRNpyler [29] in Python offer ODE based modeling of biological systems for the users who are experienced and/or interested in coding. In platforms like VCell (https://vcell.org/) and Morpheus [30], however, a user does not have to actively code but can still analyze differential equation systems [31].

Blucher *et al.* use a similar ODE system to Eqs. 1–3 to model integrin–ligand binding kinetics. They use rate constants that are consistent with values measured for multiple types of integrins by atomic force microscopy [32], and estimate the initial values for the concentration of each species. In this sense, this model can provide only theoretical information on the reaction kinetics. They solve the equations in MATLAB by both deterministic and stochastic simulations. When averaged, the results of 100 stochastic simulations matched the deterministic simulations. Moreover, they reveal that the system is most dynamic during the first quarter of the simulation time and then reaches a steady state for each model species [33].

Each of the 24 different integrin molecules goes through the processes of activation, ligand binding and clustering at different rates. In their model, Blucher *et al.* do not take into account the different integrin and ligand types. They rather provide a general overview of the dynamics of ligand binding to integrins. Although it is still interesting to mathematically explain the interactions at the cell–ECM interface, adding the complexity of different integrin–ligand pairs to such an ODE system would provide more biologically relevant estimations. This of course requires knowledge of different reaction rates for different integrin–ligand pairs, which is not present for all (see discussion for more details).

Besides being dependent on the types of interacting molecules, integrin–ligand binding is a dynamic process and is greatly affected by the changes in ECM. Proteomics studies demonstrated that ECM composition dynamically changes in response to acute stress of injury [8], [34]. The effect of changes in the ECM ligand concentration on the integrin binding kinetics can be quantified using an ODE model similar to Eqs. 1–3, when the ligand and integrin concentrations are known experimentally. Hudson *et al.* addressed this question using a combined *in vivo*–*in silico* approach, including a mouse model to induce fibrosis in the liver with CCl_4_ exposure and a complementary *in silico* model. Using liquid chromatography with mass spectrometry (LC-MS/MS) quantification, an increase in the amount of multiple types of collagen, fibrillary proteins, glycoproteins and proteoglycans in case of chronic CCl_4_-induced liver fibrosis was reported. Next, an ODE model to quantify the changes in integrin–ligand binding and integrin clustering upon changes in the ECM composition was used. The ODE model is similar to the simple example above except that Hudson *et al.* account for clustering among integrin molecules as well and assume all the integrins are active by the time the simulation starts. This is a valid simplification because this model is focused more on the integrin–ligand binding kinetics and accounts for different binding rates of different integrin–ligand pairs, which increases the number of equations to be solved.

**Fig. 3:**
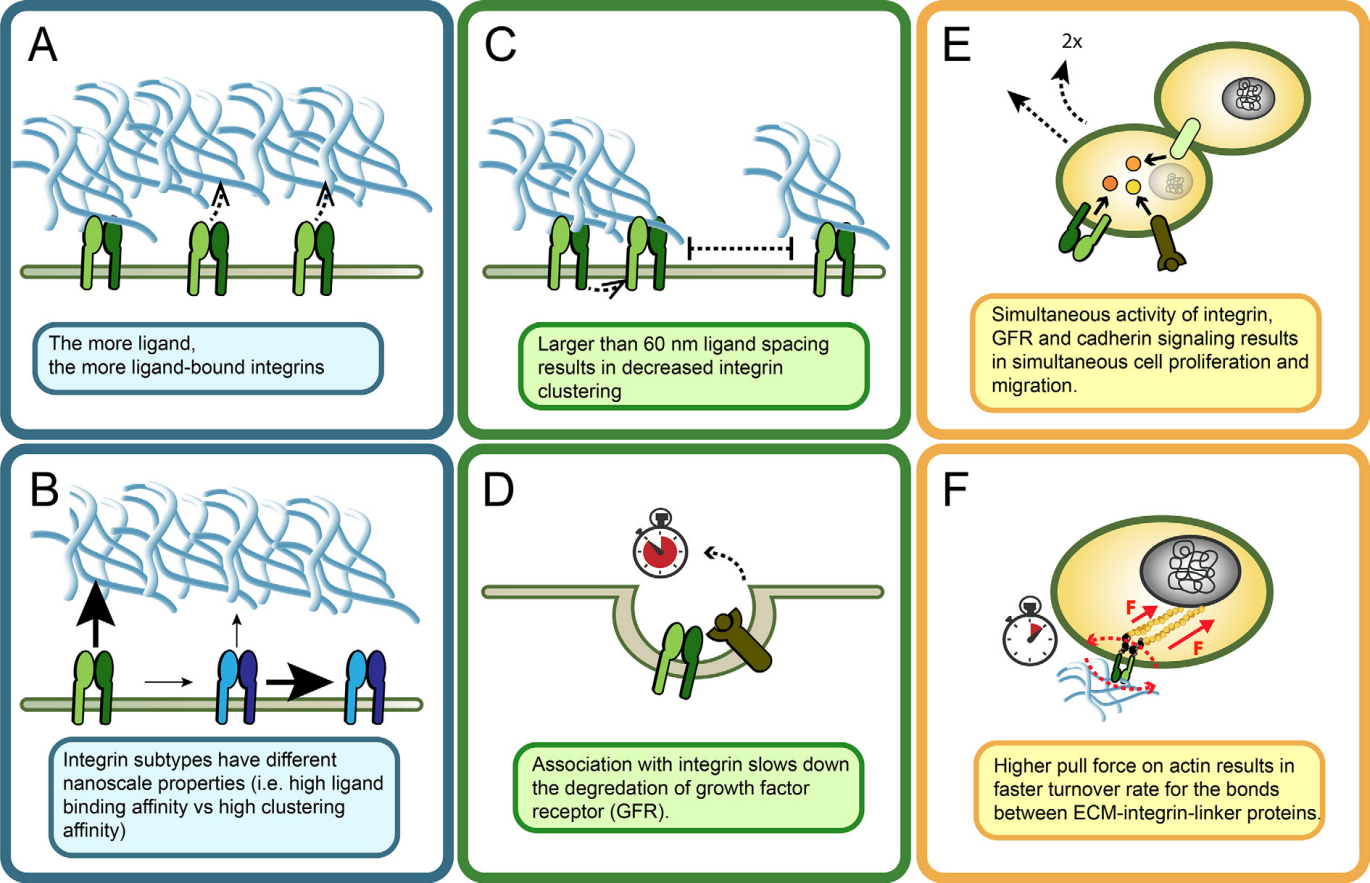
Graphical illustration of main findings from computational models about integrin function in three cellular levels.

The dynamics of integrin type α_1_β_1_–collagens type I–IV and integrin type α_V_β_3_–fibronectin and von Willebrand factor are investigated separately by Hudson *et al..* For all integrin–ligand pairs in this study, when the ligand amount increases — as in case of fibrosis in the tissue — the steady-state value of ligand-bound integrins is higher and is reached faster than in healthy tissue conditions (Fig. 3.A). When expanded to account for further behavioral effects of integrin signaling on the cell, which will be discussed in the following sections, this model could provide valuable information on the timing of events for different integrin types at the cell–ECM interface.

Although individual integrin–ligand pairs can be modeled and simulated mutually exclusively, as by Hudson *et al.,* multiple different integrin types are found simultaneously at the cell–ECM interface. The reasons for this coexistence of different types of integrins at the adhesion sites have been widely discussed. Different nanoscale properties of integrin subtypes point to different roles for them. β_1_ integrins, for instance, are found basally active on the cell membrane whereas β_3_ integrins rapidly transform between active–inactive states [35]. Similarly, β_1_ integrins have higher affinity for fibronectin than β_3_ integrins do [21]. Explaining the contribution of the different integrin properties, such as activation and ligand-binding affinity, to the adhesion formation is a challenge *in vitro* as well as *in silico*.

Using a highly coarse-grained model, Bidone *et al.* demonstrated that the simultaneous presence of β_1_ and β_3_ integrins at the nascent adhesion sites can in fact contribute to different aspect of the physics of cellular adhesion. The model space is a quasi-2D surface on which single-point integrins can get activated/deactivated, bind to ligands and interact with each other to form clusters. Integrins that have high affinity for a ligand and that are also more stable in their active state — such as β_1_ — are responsible for strong individual adhesion to extracellular ligands. Conversely, integrins with lower affinity for a ligand — such as β_3_ — have stronger affinity for other integrin molecules, resulting in focal adhesions where many integrins cluster but their binding to the ECM ligands is less consistent than for β_1_ integrins [21]. Their work therefore suggests that integrin–ligand binding and integrin clustering are competing processes and that the nanoscale properties of integrins determine the dominant process (Fig. 3.B).

The computational models mentioned here either considered integrins as equally active and ready-to-bind to ligands [8] or as molecules with a defined rate of activation [21], [33]. This was because these models were focused more on the chemical reactions that occur during or following the integrin-ligand binding, therefore a more detailed representation of the integrin activation was not necessary. However, there is a significant amount of computational modeling efforts around the actual mechanisms of integrin activation. These efforts mainly include molecular dynamics simulations, and although out of the scope of this review, we refer the readers to [36], [37] for two of such molecular dynamics simulations.

## 3 Integrin function on the cell membrane

The second spatial level of integrin action is on the cell membrane where integrins have two main function with long range effects: 1) clustering among each other and 2) cross–talk with other cell membrane receptors. Clustering of ligand–bound integrins happens with the help of polyvalent extracellular ligands and/or cytoplasmic linker proteins and is prominent in the maturation of focal adhesions [38]. The cross-talk between integrins and other cell membrane receptors is important in establishing and maintaining intercellular signaling cascades that have further impact on cell behavior [14]. As such, it is interesting to explore the mathematics behind the molecular biology of integrin activity on the cell membrane.

### 3.1 Integrin clustering

Experimental observations suggest the joint effect of substrate stiffness and spatial organization of ECM ligands to be prominent in the formation of cellular adhesions. Substrate rigidity regulates the dynamics of cell adhesion by modulating the mechanical force to promote conformational changes in integrin and ligand molecules [39], [40]. Therefore, rigidity influences the reactions between integrin and their ligands, which were discussed in the previous section. The spatial organization of integrin ligands, however, is observed to affect the continuance of the cellular adhesion which is related to clustering of ligand–bound integrins on the cell membrane. On substrates where the ligand spacing is larger than ~ 70 nm, the focal adhesions stay immature (because integrins cannot cluster) whereas ligand spacing smaller than ~70 nm promotes maturation of focal adhesions [40], [41].

The underlying mechanochemical mechanism of integrin clustering in response to ligand spacing has been studied by Yu *et al.* using Monte Carlo simulations. Their model space was a ~6 × 6 µm^2^ square patch of cell membrane with ~100–1000 integrins that are free to diffuse to the non-occupied, nearest-neighbor location. The model does not account for different types of integrins but rather simulates the integrin function in a generalized way. At each step of the simulation, integrins follow a set of rules based on a system that is similar to the one introduced before (Rxn 1–2), except that integrin clustering (Rxn. 3) is added to the reaction system and the ODEs are rearranged accordingly.

(Rxn. 3) $$\mathrm{LI}+LI\overset{k_{C}^{+}/k_{C}^{-}}{↔}C$$

According to reaction (3), ligand-bound integrins can form clusters (C) with the nearest ligand-bound integrin (LI) at rate $k_{C}^{+}$, and similarly, clusters can dissociate at rate $k_{C}^{-}$. The Monte Carlo method introduces stochasticity to the system at the beginning of each simulation by randomly sampling the integrin molecules on the membrane that will undergo reactions (1), (2), (3) and therefore setting the initial conditions for the ODE system. Stochasticity is inherently present in biological systems since many cellular reactions depend on the random motion of molecules [42], so it is important to account for the randomness. The integrins that are not contributing to the ODEs are free to diffuse on the membrane to the next nearest neighbor with a constant rate. This way, the integrins are shuffled at each simulation step and their spatial distribution changes [43].

In another interesting *in silico* experiment, Yu *et al.* set ligands at fixed positions in the model space, but with different spacing between them (20, 40, 60, 80 and 100 nm). At the end of the *in silico* experiments with ligand spacing 20 to 60 nm, 60% of total integrins in the model space are found in the clustered form. With ligand spacing exceeding 60 nm, a sharp decrease in the amount of clustering is observed (Fig. 3.C). At 80 nm ligand spacing or greater, only about 10% of integrins are clustered [43]. The reason behind this observation is that when ligand spacing is larger than a threshold of 60 nm, there are less ligands available (L in Rxn. 2) in the designated experiment space for the active integrins (I in Rxn. 1 & 2) to bind while diffusing through the cell membrane, therefore reducing the possibility of integrin clustering (Cin Rxn. 3). Consequently, these *in silico* results provide a mechanochemical mechanism of the experimental observations that ligand spacing is crucial in adhesion maturation [40], [41], [43].

Another *in silico* model has achieved a similar result. In their agent-based model (ABM), Jamali *et al.* conclude that ligand spacing has a key role in initiating integrin clustering. More specifically, in an ABM, each element in the system is called an agent and agents interact with each other following particular rules of interaction (i.e. biochemical reactions). Different from ODE-based models, to account for the heterogeneous and stochastic nature of biochemical systems, ABM models assign a certain probability when assessing each interaction between the agents, rather than assigning a particular rate for each reaction [44]. The assigned probabilities of events are calculated based on the observed properties of agents. For example, the agents move to a new location within the model space with a probability that correlates with the actual diffusion coefficient of the molecule each agent represents [44].

The ABM model of integrin clustering also shows that increased affinity between integrin subunits promotes clustering especially when ECM ligand concentration is low [44]. However, both of the models report for a standardized integrin–ligand pair and do not comment on the effects of different reaction rates when considering different integrin–ligand pairs. Yet both models could be made integrin- and ligand-type specific, when the binding energies required for a specific integrin–ligand and integrin–integrin pair would be known.

An interesting remark by Yu *et al.* is that integrin clustering might not only be orchestrated from the ECM side but also from cytosolic side. This is based on the experimental findings of integrin activation being accompanied by talin binding to the β subunit of integrins. Talin is a protein forming the link between integrins and the cytoskeleton and it has also been shown to aid integrin clustering via its head domain [16], [43], [45]. Therefore, it is exciting to hypothesize that integrin clustering on the membrane is tightly controlled by the ligand organization on the ECM side, but also affected by the events happening in the cytosol, although further research is needed here.

After observing that integrin clustering is mediated by ECM properties and cytosolic factors, an anticipated question is about the role of these integrin clusters in the process of mechanotransduction. A recent spatial Monte Carlo model by Cheng *et al.* suggests that large integrin clusters on the cell membrane are spots for focal adhesion kinase (FAK) phosphorylation. As the substrate stiffness affects the size of the clusters, it directly translates into the level of FAK phosphorylation, hence downstream signaling. They validate their model findings with *in vitro* experiments and show that stiffer substrates result in larger integrin clusters and more phosphorylated FAK is found in cytosol [46]. This model provides an explanation for how different cell types respond to different ECM stiffness and proposes different roles for different integrin types, in accordance with Bidone *et al.* model from 2019 [46].

### 3.2 Cross-talk between integrins and other membrane receptors

Being on the cell membrane, integrins are known to cooperate with other cell membrane receptors to serve their crucial role in regulating biological events, like cell migration and proliferation. Transforming growth factor beta (TGF-β) receptor, platelet-derived growth factor (PDGF) receptor, vascular endothelial growth factor (VEGF) receptor, bone morphogenic protein 2 (BMP-2) receptor are, for example, known partners of integrins [14], [47].

Experimental observations indicate the stabilization of VEGF receptor activity upon interaction with integrin α_v_β_3_ during angiogenesis. It is also known that a protein kinase, Src, is a mediator between the two receptors [15]. Yet the exact mechanism of action of this cooperation could not be revealed by *in vitro* methods. A kinetic rule-based ODE model by Bazzazi *et al.* investigated the mechanisms behind this cross-talk.

The *in silico* model of Bazzazi *et al.* consists of four species: integrin α_v_β_3_ as a single entity, VEGF receptor, VEGF and Src kinase. Integrin and VEGF receptor are assumed to be pre-associated with each other in an inactive state in order to avoid the complication of modeling the physical proximity of the molecules. The following set of rules defines the biological actions in the model: 1) VEGF binding to VEGF receptor, 2) VEGF receptor activation by autophosphorylation, 3) internalization and degradation of VEGF-bound VEGF receptor, 4) activation of Src, 5) integrin activation by phosphorylation by Src, and 6) active integrin–active VEGF receptor association at a second site. They obtain the parameter values by fitting the model to a consistent set of experimental studies and further perform a sensitivity analysis to identify the most sensitive parameters and thereby the most essential step in the reaction set [48].

From the global sensitivity analysis, the rate of internalization and degradation of the VEGF receptor is approximately 400-fold lower when it is associated to the active integrin than when they are not associated. In other words, the underlying mechanism of stabilization of VEGF receptor activity by integrin is via slowing the degradation process of the VEGF receptor (Bazzazi et al. 2018, Fig. 3.D). This model cannot be generalized to every integrin and associated receptor, but it is one of the few attempts in explaining the mechanism behind cross-talk between integrins and other receptors in angiogenesis and is therefore of great value. Another *in silico* model deals with receptor cross-talk but is focused on the interactive effects of the two receptors in downstream signaling, rather than the receptor-integrin kinetics [49]. Therefore, it will be discussed in the appropriate section below.

## 4 Integrin function in the cytosol

The third level of integrin function is in the cytosol, where external cues are translated into biochemical signals. Integrin adhesomes, complexes harboring multiple proteins, assemble at the cytoplasmic tails of integrins as the adhesions mature. The composition of the integrin adhesome is cell type specific, however, with an attempt to identify a consensus set, 60 proteins have been found to be crucial in the integrin adhesomes [50], [51]. Components of integrin adhesome are responsible for transmitting the signals received from integrins to other parts in the cell, eventually affecting cellular decision-making. There is extensive literature on the molecular biology of signaling pathways where integrins are involved as well as their effect on cell behavior [45], [52]. Here, we focus on *in silico* methods that quantitatively explore the action mechanism of cellular events in response to integrin function.

### 4.1 Signaling activities

Active and ligand-bound integrins, via the multiprotein complexes at their cytoplasmic tails, are known to activate focal adhesion kinase (FAK) and start a signaling cascade that affects cellular behavior, such as motility or proliferation. Players of the growth factor receptor and integrin cascades interact by inhibiting/activating one another in feedback loops. The details of these signaling cascades are out of the scope of this review and can be found in other dedicated reviews [3], [45], [52]. In the next paragraphs, we review the *in silico* models of integrin signaling and cross-talk.

The association of integrins and growth factor receptors in angiogenesis was mentioned earlier as we explored integrin-VEGF receptor (VEGFR) association that stabilizes VEGFR activity by slowing the receptor degradation [48]. Growth factor binding to growth factor receptors (GFR) promotes proliferation and cell survival mostly via the mitogen-activated protein kinase (MAPK) signaling pathway. However, growth factor–initiated signaling is not enough for the cell to proliferate. It is also known that cell-to-cell communication via cadherins is another factor that ensures proper cell proliferation in presence of growth factors in angiogenesis [53]. These findings suggest cross-talk between the signaling pathways of three cell membrane receptors, namely integrin, VEGFR and cadherin.

Bauer *et al.* explore the interplay between downstream signaling to VEGFR, cadherin and integrin during angiogenesis, with a stochastic Boolean network model. In Boolean networks, molecular species show a binary behavior, i.e. they are either ON or OFF. It is a practical way of modeling when the quantitative kinetic data of particular biochemical reactions is not enough to support an ODE type of model. If the concentration of a molecular species in the model at any time point is above a certain threshold, the species is ON, while the concentrations below the threshold translates into OFF behavior. A “probability of happening” function is added for each molecular interaction in the Bauer *et al.* model, to account for the noise or randomness in signaling cascades.

To test the dependence and additive effects of VEGFR– and integrin–induced signaling while also taking into account the cell–to–cell communication through cadherin signaling, they set the initial states of molecular species to randomly chosen binary sets (ON/OFF) and report the output states that correspond to the following cell level phenotypes: proliferating, quiescent, migratory and apoptotic. As expected, in the absence of VEGFR or integrin signaling activity, apoptosis is induced. When VEGFR and integrin signaling are both active, they observe one signaling molecule, namely Rac, is of particular importance. In their model, active Rac enables the cells to migrate whereas inactive Rac results in quiescent cells. When they allow cell-to-cell contact together with active VEGFR and integrin signaling and Rac activity, proliferation is observed while cells migrate (Fig. 3.E). While this is contrary to the general assumption of proliferation and migration events being temporally exclusive of each other [54], [55], [56], there is evidence of both events happening simultaneously in cancer cells [57]. Wound healing is another process, that requires both cell motility and proliferation, and the predictions from the Bauer *et al.* model might be helpful in that area of biology [58].

Another *in silico* model explores the effect of Rac and related signaling on protrusion velocity at the leading edge of motile cells [59]. It is an ODE-based model with stochastic simulations that reports the protrusion velocity in a dimensionless manner. This model incorporates the modulation of Rac signaling by nascent adhesions via paxillin, which is one of the proteins in the complex that interacts with integrin cytosolic tails. The Cirit *et al.* model also confirms the positive correlation between Rac activity and cell protrusion in case of nascent adhesion between the cell and the ECM.

In addition to modeling the cellular response to integrin-related signaling, the dynamic assembly-disassembly of the integrin adhesome is another area in biology that can benefit from computational methods. Exploring the composition of the adhesomes at the time of assembly and disassembly via proteomics analyses revealed that integrin presence is stable throughout the assembly-disassembly (30 min). However, adaptor proteins between integrins and actin cytoskeleton leave the assembly in about 15 min and with different kinetics [50]. The distinct binding-unbinding kinetics of adhesome proteins have been studied experimentally using techniques like fluorescence recovery after photobleaching (FRAP) [60], [61]. Computational modeling can help to interpret these experimental findings by simulating various hypotheses on the assembly and disassembly of the integrin adhesome, complementing the previous work on functional network identification of the integrin adhesome [50], [51], [62], [63]

### 4.2 Cytoskeletal remodeling

Integrin and downstream signaling activities directly affect cell motility as summarized above. Yet, the physical connection of integrins to the actin cytoskeleton (Fig. 1) is crucial for the cell to actively change its shape to accommodate motility. During protrusion, the actin cytoskeleton actively changes shape and length. Actin-linked integrins are subjected to myosin-mediated forces while still being linked to the ECM proteins on their ectodomains. At this point, when integrins are already bound to their ligands and to the actin cytoskeleton, understanding the effect of push and pull forces on integrins is as important as understanding the effect of ECM properties on the cell shape and migration. For this reason, most of the computational efforts on modeling of the cytoskeletal remodeling and cell shape changes focuses on the mechanics of the process, and some of these efforts have been nicely reviewed [27]. Here, we mention two *in silico* models that are at the molecular level and focus more on the biochemical reaction dynamics. Importantly, both of these *in silico* models do not treat integrins as individual entities but either as a complex always bound to its ligand [64] or as an inseparable part of a bigger adhesion complex [65].

The ODE-based model by Macdonald *et al.* considers the binding–unbinding events between three main species: actin filaments, integrin receptor bound to a ligand on the ECM and a linker complex that theoretically contains all the proteins linking the integrins to the actin cytoskeleton. All three species can combine to form complexes of 2 or 3 among them. The effect of the force exerted on the linkages between the integrin–linker complex and actin is modeled in two ways: 1) negatively by increasing the dissociation rate constants of all possible complexes 2) positively by reinforcing the bond between the species in the actin–linker–integrin complex. This opposing effect of force on linkages creates a biphasic scenario when steady-state levels of each species are observed at changing levels of force (10^-12^ to 10^-10^N). Lower levels of force exerted on actin/integrin linkages results in a higher number of actin–linker–integrin complexes than when under high force levels. A force of 10^-10^N causes the breakage of linkages, while a force of 10^-12^N strengthens the linkages [64] (Fig. 3.F).

A rather more sophisticated model of actin remodeling is described by Escribano *et al.* They modelled the ECM with a discrete number of ligands on it, the adhesion complex that is representative of the integrin and the linker proteins in the cytosol, the actin filaments with changing length and the myosin motor proteins of which the number affects the amount of the pull force. At high levels of pull force (i.e. high number of myosin proteins on the actin filaments), this *in silico* model proposes an increased velocity for the actin filaments [65]. This is in accordance with the previous model of MacDonald *et al.,* as increased velocity requires increased turnover rate for the bonds between adhesion molecules and the ECM.

As force measurements at the cellular level are better established to date than measurements at the single molecular level (that are required to measure rates of integrin clustering, for example), these type of models have a bigger pool of experimental data for validation (see also discussion section). Findings of both models described here have been validated experimentally [66], [67].

## 5 Conclusion and outlook

As understood from the many different methods mentioned here, there is no single truth when it comes to computational modeling of integrin function. There are multiple ways to model integrin function, each with their advantages and disadvantages, and often determined by the particular research question. In fact, this is valid for all attempts to computational modeling of biological systems. The level of complexity and nonlinearity in biological systems make it necessary to reduce the system to its essential components (and thus to simplify), while the ever growing possibilities in mathematical and computational methods create new avenues of exploration.

The computational models that address integrin function up to date, capture —to some extend—the nature of events at the cell-ECM interface, albeit focusing on particular processes at distinct scales. In silico models of the binding between integrins and their ECM ligands provides an understanding of the binding kinetics and reflect how the kinetics can change with changing ECM conditions [8]. These models, usually expressed as ODEs, fall short on spatial aspects of ligand binding as they do not factor in the spatial variations. Models of integrin clustering, provide an explanation for the experimental observations that ligand spacing is a limiting step in the process of integrin clustering [43], [44]. Yet the model geometry is always a rather simplified “square” or “round” area. The majority of computational studies on integrins have captured integrins in general, without specifying the particular subtypes. Simplifications have also been made about the interaction partners of integrins. Models of integrin–ligand binding assume integrin binding to one ligand at a time whereas in reality one integrin has affinity for binding to multiple ligands in the ECM, resulting in ligand binding competition. On the cytosolic side, large signaling pathways have been simplified to Boolean network models that account for key molecules such as Rac [49] whose activity can explain certain phenotypes. Yet, these types of models lack the numerical details ODE type of models can provide, and in particular, the kinetic rates of the underlying processes. Altogether, *in silico* models of integrin function, provide mathematical explanations to the interactions between integrins and their ligands, integrins and other cell membrane receptors and integrins among themselves.

In this review, we highlighted the *in silico* models that focus on the chemical reaction dynamics of integrin-related cellular events. However, as integrins are “physical anchor” points between the ECM and the cytoskeleton, they are central to mechanosensitive cellular processes. These include the cellular responses to external mechanical cues like increased proliferation and motility on stiff substrates [7]. In order to explain the roles of integrins in mechanotransduction, computational models have been developed from a more mechanical point of view as well. For example, Chan and Odde 2008, introduced the “molecular clutch” model of focal adhesions and explained the biphasic behavior of filopodia in response to mechanical stiffness of the environment [66]. Molecular clutch model represents the engagement between the ECM and the actin cytoskeleton, which resists actin retrograde flow powered by myosins. On stiff substrate, low traction force results in high retrograde flow thus increased protrusion. On the contrary, on soft substrate, high traction force reduces the actin retrograde flow and the protrusion rate of the filpodia [20], [66]. This molecular clutch behavior has been adapted by many others and further developed. Ligand-bound integrin molecules (i.e. clutches) have been usually modeled as springs and as a part of a bigger architectural complex in these purely mechanical models. Using the molecular clutch principle, Oria et al. have explained the link between adhesion formation and rigidity-dependent ligand spacing sensing of the cells [68]. We refer the reader to [69] for a nice overview of the molecular clutch hypothesis.

To further the field of integrin biology, a crucial next step, in our opinion, is to combine the computational models that focus on chemical reaction networks with mechanical models. An example of such initiative is the work of Shuaib et al. where they introduced the concept of a hybrid mechanical-agent-based model for bone tissue mechanotransduction. The agent-based model predicts the cytosolic production of ECM proteins, influenced amongst others by the mechanical and compositional inputs of the ECM. These compositional changes of the ECM alter the properties of the mechanical model, which in turn affects the input parameters for the agent-based model [70]. Such hybrid models are promising, yet can be challenging due to their multi-scale nature. The next paragraphs detail the next steps for modeling the function of integrins computationally, both from a biological perspective as a technical point of view including the challenges of multiscale and multiphysics modeling.

### 5.1 Overcoming biological challenges

Modeling the behavior of different types of integrins is one area that is open for exploration. Only four of the models mentioned here (namely; [8], [21], [46], [48]) account for the integrin subtype-specific ligand binding or clustering properties. However, a deeper understanding of differences between integrin subtypes will be helpful in understanding their distinct roles at the cell-ECM interface and therefore cell type-specific integrin expression [10]. Related to this, the competition of multiple ligands with affinity for binding to the same integrin type (Fig. 2) represents an important model extension as it allows to understand the reasons of various ECM compositions per tissue and to reverse engineer synthetic matrices (see also below) [71]. Another area of exploration that is biologically relevant is the cross-talk between other cell surface receptors and integrins. For example, it is known that integrins play a central role in activation of TGF-β in ECM, but the interactions between the TGF-β receptors (an RTK) and integrins have not been fully understood [72], [73]. Another example of integrin cross-talk is with syndecan receptors. Syndecans are transmembrane proteins which often serve as coreceptors, for example by recruiting ligands for other receptors [74]. Especially cross-talk between syndecan-4 and integrins is shown to enhance interactions between the ECM and cytoskeleton [75]. Computational modeling could help unravel the underlying mechanism of action in this cross–talk.

In terms of extending the biological scope of the models, one major challenge in modeling integrin subtypes is in obtaining accurate quantitative measures (i.e. the parameters for the model) on each subtype. It is generally difficult to isolate and quantify transmembrane proteins intact and in desired conformations and integrins are not an exception [2], [76]. Therefore, for ODE models that require absolute concentration of inactive and/or active integrins on the cell membrane (e.g. Equations (1), (2), (3), the limiting step is obtaining these dynamic quantitative measures. To tackle this challenge, experimental scientists apply indirect ways of measuring the density of integrins on the cell membrane. For instance a good example is by Elosegui-Artola *et al.* 2014, where they measure the fluorescence intensity of cells when they are bound to rhodamine-labeled fibronectin via integrins on the cell surface. The fluorescence level is then converted into concentration, using the level of emitted fluorescence by known concentrations of fibronectin for their experiment. Another challenge, especially on the way to increase the specificity of the computational models in terms of integrin–ligand pairs, is measuring integrin–ligand binding/unbinding rates. This usually requires sophisticated techniques like surface plasmon resonance (SPR) [77], [78], [79] or single molecule dynamic force spectroscopy [80]. These techniques are not available to all molecular biology labs and require operational expertise as well as very technical equipment. There is an evident need of collaboration between computational and experimental scientists to unravel the unknowns of integrin function.

We propose for the case of computational models of integrin function, experimental biomaterial design is a field where the complementary model–experiment cycle can be established and maintained. In particular, by using modular, synthetic materials, the influence of distinct microenvironment components (e,g. mechanical information, (fractions) of ligand types and ligand concentration) on integrin binding can be assessed individually [81]. Also, these precisely-defined, tunable materials allow for the measuring of binding strength of specific integrin–ligand pairs by SPR, since the ligands could be isolated and exactly controlled in concentration. Synthetic supramolecular assemblies have great promise for this, because their monomeric building blocks could be functionalized with bioactive cues to easily introduce function using a modular approach [82], [83]. Noteworthy, the type of supramolecular base material that is used to present the integrin-binding supramolecular additives (i.e. RGD or cyclic (c)RGD conjugated to the corresponding supramolecular motif) affects cell adhesion properties; a bisurea (BU)-based material presents the additives more effectively over a ureido-pyrimidinone (UPy)-system [84]. Also the ligand concentration influences integrin-binding properties, as an increasing concentration of accessible cRGD leads to more FA formation with a decreased size. Furthermore, ligand type effects integrin targeting, since different ligands contain different binding affinities for certain integrin dimers [85]; it was shown that substrates containing the higher affinity ligand cRGD led to a two times higher cell attachment rate and had twice the number of FAs than the cells cultured on substrates with its linear equivalent. Another example of modular, integrin-targeting materials is synthetic peptide amphiphiles (PAs) as pioneered by the research group of Stupp. Here they for example employed bioactive PAs bearing the fibronectin-derived RGDS-motif as scaffold for stem cell delivery [86]. Next to this, Mardilovich et al. designed PAs decorated with fibronectin-derived integrin-binding motifs GRGDS and its synergistic PHSRN sequence in a spatially controlled manner that matched the natural distance found in fibronectin [87]. They observed similar cell behavior for the synthetic PAs as for the natural fibronectin, and even stronger FA formation and reorganization of the cytoskeleton was found for the PAs. This highlights the importance of using integrin-binding materials with matching spatial organization to its natural counterpart for effective integrin binding. On this note, another class of supramolecular biomaterials in which the spatial organization of integrin-binding motifs can be controlled precisely is DNA origami, owing to its robustness and programmability [88]. To illustrate, Palma et al. designed and synthesized a multi-ligand functionalized, nanoscale particle containing spatially controlled A20FMDV2, an integrin αVβ6-binding motif, and epidermal growth factor (EGF), a protein that binds the epidermal growth factor receptor (EGFR) which is a tyrosine kinase that cooperates closely with integrins [89]. They showed that a ligand spacing of 60 nm and the presence of 3 peptides, so 3 integrins, led to maximum cell attachment. Altogether, these examples illustrate the suitability of tunable, modular biomaterials to isolate and judge the effects of distinct microenvironment elements (e.g. ligand concentration and type as well as spatial organization of ligands) on integrin-binding.

In summary, to push forward the field of integrin biology, we invite the field of biomaterial design and *in silico* modeling to come together and think about relevant biological questions and hypotheses to unravel in an iterative loop of simulation and experimental validation [25]. On one hand, *in silico* models could help in predicting the performance of biomaterials which are suitable in steering a desired cellular outcome. In this way, not the full library of materials is required to be synthesized and assessed, but only a relevant range, thereby minimizing research time, effort and costs. While on the other hand, it is the experimental side that could complement *in silico* integrin models, by both providing input values (e.g. binding rates between ligand–integrin receptor) for the computational integrin models and by serving as validation for the model outcomes. In this way, the experimental and computational worlds on integrins should come to a closed cycle that complete one another.

### 5.2 Overcoming computational challenges

Finally, we turn to our perspective towards what lies ahead for the computational field. In particular, there are three areas where we stand to make significant progress. Firstly, to fully understand and help unravel the biology of integrin function, it is essential, in our opinion, to computationally integrate all three layers of action spatially and temporally. In the current computational models, we obtain separate snapshots of events happening at the three distinct spatial layers. However, to be able to simulate and predict all the mechanical interactions and chemical reactions happening, starting with the binding of integrins to the ECM ligands up to the cell’s behavioral reaction (e.g. proliferation, differentiation, apoptosis etc.), we will need computational models that combine the mechanochemical integrin actions at the three spatial levels. Modeling the biochemical processes and the mechanical responses of the cytoskeleton as well as the complex mechanochemical feedbacks that emerge from integrin signaling is challenging due to, amongst others, the following technical challenges:

1) The biochemical reactions occurring downstream of the integrin receptors are fundamentally stochastic in nature (e.g. small copy numbers), resulting in local gradients and heterogeneities. Although various algorithms exist for stochastic simulations [90], [91], they become computationally intractable for large chemical reaction networks with many species;
2) Eukaryotic cells consist of three main kinds of cytoskeletal filaments: microfilaments (actin, 7 nm diameter), microtubules (tubulin, 25 nm diameter) and intermediate filaments (various proteins, 12 nm diameter). Ideally, one would like to represent the exact filamentary network, meaning that all three types of individual fibrils must be discretized at sufficient resolution to resolve the biochemical reactions with the cytoskeleton as well as calculate an accurate mechanical force field (which in turn results in remodeling of the cytoskeletal network);
3) The interesting mechanical and biochemical phenomena take place at the nanometer scale whereas the emergent behavior occurs at the micrometer scale. As such, there is a need to scale-up the simulations in a computational efficient way while retaining the required spatial resolution;
4) Integrin–ligand binding occurs within seconds whereas adhesion maturation requires minutes. Downstream events in the cytosol, from signaling and actin cytoskeleton reorganization to cellular differentiation, can take hours to days to weeks. Similarly to the spatial scale, systems with reactions that operate at very different time scales require advanced numerical methods since otherwise every single fast reaction would need to be simulated, requiring a huge computational effort [26].

Much progress has been made, including optimized numerical algorithms to efficiently solve sparse reaction–diffusion networks [92], [93], software packages to simulate active cytoskeleton network dynamics [94] and advanced hybrid and multiscale techniques to couple various spatial and temporal scales [95], [96]. The idea behind the hybrid and multiscale techniques is to partition the system into different spatial or temporal scales and then apply different simulation methods that better fit the scales (e.g. stochastic at the intracellular level, deterministic reaction–diffusion at the tissue level). However, as the partitioning and linking (after the simulation step) introduces errors, it is necessary to develop advanced methods that allow linking different scales in an accurate manner.

Secondly, we are in the age of parallel computing with advanced (parallelized) numerical methods to fully leverage this power. Parallelized calculations can be done on a multicore desktop computer, on high-performance clusters or using cloud computing services. The new hardware developments, including advanced graphical processing units (GPUs) are rapidly increasing the computational power. At the same time, many software plugins are becoming available for Matlab [97], Python [98], etc. to exploit the power of graphical processing units (GPUs), greatly reducing the computational time.

Thirdly, due to the nature of computational studies - written in a computer-readable coding language - it is possible to easily extend existing *in silico* models or to combine elements of different models to create a new model with larger spatial and temporal scope. However as in experimental studies, for a computational model to be revisited and potentially extended by other scientists than the original authors, the model should be reproducible. There exist many exciting initiatives such as model software repositories (e.g. VCell DB [https://vcell.org/], BioModels [https://www.ebi.ac.uk/biomodels/], CellML repository [https://www.cellml.org/]) and collaborations between publishers and the Center for Reproducible Biomedical Modeling (https://reproduciblebiomodels.org/) to check, increase and maintain reproducibility at the peer–review stage in publication process [99].

All in all, we believe such practices will help the computational biology field to become more accessible and that through *in silico-in vitro* collaboration we will gain a great amount of fundamental knowledge on integrin biology.

## CRediT authorship contribution statement

**Zeynep Karagöz:** Conceptualization, Visualization, Writing - original draft, Writing - review & editing. **Laura Rijns:** Writing - original draft, Writing - review & editing. **Patricia Y.W. Dankers:** Writing - review & editing, Supervision, Funding acquisition. **Martijn van Griensven:** Conceptualization, Writing - review & editing, Supervision. **Aurélie Carlier:** Conceptualization, Writing - original draft, Writing - review & editing, Supervision, Funding acquisition.

## Declaration of Competing Interest

The authors declare that they have no known competing financial interests or personal relationships that could have appeared to influence the work reported in this paper.
